# Design of a Multicomponent Peptide-Woven Nanocomplex for Delivery of siRNA

**DOI:** 10.1371/journal.pone.0118310

**Published:** 2015-02-23

**Authors:** Eunsung Jun, Soyoun Kim, Jong-Ho Kim, Kiweon Cha, In-Seop So, Hye-Nam Son, Byung-Heon Lee, Kwangmeyung Kim, Ick Chan Kwon, Sang Yoon Kim, In-San Kim

**Affiliations:** 1 Department of Biochemistry and Cell Biology, Cell and Matrix Research Institute, School of Medicine, Kyungpook National University, Daegu, Republic of Korea; 2 Department of Pharmaceutical Science, College of Pharmacy, Kyung Hee University, Seoul, Republic of Korea; 3 Division of high-risk pathogen research, Korea National Institute of Health, Korea Centers For Disease Control & Prevention (KCDC), Osong, Chungbuk, Republic of Korea; 4 Center for Theragnosis, Biomedical Research Institute, Korea Institute of Science and Technology, Seoul, Republic of Korea; 5 Department of Otolaryngology, University of Ulsan College of Medicine, Seoul, Republic of Korea; University of Helsinki, FINLAND

## Abstract

We developed and tested a multicomponent peptide-woven siRNA nanocomplex (PwSN) comprising different peptides designed for efficient cellular targeting, endosomal escape, and release of siRNA. To enhance tumor-specific cellular uptake, we connected an interleukin-4 receptor-targeting peptide (I4R) to a nine-arginine peptide (9r), yielding I4R-9r. To facilitate endosomal escape, we blended endosomolytic peptides into the I4R-9r to form a multicomponent nanocomplex. Lastly, we modified 9r peptides by varying the number and positions of positive charges to obtain efficient release of siRNA from the nanocomplex in the cytosol. Using this step-wise approach for overcoming the biological challenges of siRNA delivery, we obtained an optimized PwSN with significant biological activity *in vitro* and *in vivo*. Interestingly, surface plasmon resonance analyses and three-dimensional peptide models demonstrated that our designed peptide adopted a unique structure that was correlated with faster complex disassembly and a better gene-silencing effect. These studies further elucidate the siRNA nanocomplex delivery pathway and demonstrate the applicability of our stepwise strategy to the design of siRNA carriers capable of overcoming multiple challenges and achieving efficient delivery.

## Introduction

RNA interference (RNAi) is a natural biological system by which RNA molecules silence gene expression by inhibiting protein translation. RNAi using synthetic, small interfering RNAs (siRNAs) has powerful and broad potential as a therapeutic strategy for suppressing target proteins through sequence-specific degradation of their corresponding mRNAs. Research on siRNA delivery has made great progress in recent decades, and at least 22 siRNA-based strategies for treating disease have reached clinical trials [1–4].

Attaining the full clinical potential of RNAi, however, requires delivery systems that can overcome numerous biological barriers. The delivery vehicles should encapsulate and protect the siRNA cargo from serum proteins, transport siRNA to specific target cells and tissues, and release their cargo at the site of action in these cells. Various carriers, including lipid-based agents [5], cationic polymers [6], viral capsid proteins [7] and cell-penetrating peptides (CPPs), have been designed to surmount these challenges [8,9]. Although many carriers have shown gene-silencing efficacy *in vitro* and *in vivo*, they still present other limitations, such as instability or cellular toxicity, that prevent the full potential of RNAi from being realized in the clinic.

The main challenges associated with siRNA delivery include specific cellular binding, escape from endosomes after receptor-mediated endocytosis and dissociation of siRNA from the carrier to allow incorporation into RISC [10–12]. Intracellular pathways involved in endosomal escape and disassembly of the siRNA complex are particularly complicated hurdles. Ren *et al*. designed tumor-penetrating nanocomplexes containing myristoylated tandem peptides to negotiate the biological challenges of siRNA delivery and demonstrated that the valence of the tumor-targeting ligand and overall nanocomplex charge are important parameters in determining the activity of the nanocomplex [13]. Despite continuing efforts to develop efficient carriers, understanding of the molecular characteristics of effective siRNA-carrier complexes remains incomplete and few studies have systematically addressed the multi-step pathway of siRNA nanocomplex delivery.

Here, we describe a peptide-siRNA nanocomplex comprising multiple peptides designed to facilitate siRNA encapsulation, specific cellular targeting, escape from endosomes, and release siRNA from the complex to exert biological activity. The peptide components were interwoven with siRNA forming an approximately 200-nm nanocomplex that we term a Peptide-woven siRNA nanocomplex (PwSN). To encapsulate siRNA, we employed a simple nine-arginine cell-penetrating peptide (9r-CPP). CPPs, which consist of basic or amphipathic short peptides, represent a promising type of agent for delivering nucleotides into cells [14–16]. Although CPPs offer several advantages in terms of their biological characteristics, economics of formulation, convenience of combination, and safety compared with other substances, they lack receptor specificity and are internalized by nearly all cell types [17–19]. A typical 100–200 nm CPP-siRNA nanocomplex is expected to diffuse into tumor tissues *in vivo* through the enhanced permeation and retention (EPR) effect. However, accumulating evidence indicates that the EPR phenomenon does not occur in all tumors and numerous barriers hamper passive nanoparticle delivery into tumor tissue [20].

We have developed a receptor-specific CPP by ligating an interleukin (IL)-4 receptor-targeting peptide to 9r (I4R-9r) and adopted a stepwise approach for designing peptides capable of overcoming barriers in the intracellular delivery pathway [21]. To facilitate endosome escape we synthesized an endosomolytic CPP, a short HIV gp41-derived peptide linked to 9r (sHGP-9r), that, when blended with I4R-9r, formed a ternary complex with siRNA through noncovalent interactions. The sHGP peptide is a common fusogenic peptide that contains a motif of alternating acidic and hydrophobic residues [22–24]. To ultimately gain efficient gene knockdown, we modified 9r-CPP with different combinations of alanine substitution and evaluated the optimal 9r-variant for dissociation of siRNA from the carrier. To understand how biochemical parameters such as binding affinity and complex stability contribute to gene-silencing effects, we analyzed complex assembly and disassembly using surface plasmon resonance (SPR) spectroscopy. We also discuss the relationship between binding characteristics and biological activities based on three-dimensional (3D) structural models of peptides. This systematic, stepwise approach for more efficient gene silencing provides insight into the siRNA-delivery pathway and aids in designing optimized siRNA carriers.

## Materials and Methods

### Peptides and siRNAs

The tandem peptides were synthesized via standard FMOC solid-phase peptide synthesis and purified by high-performance liquid chromatography (Peptron Co., Ltd, Daejeon, Korea). The sequences of peptides are: I4R-9r (CRKRLDRNC-GG-RRRRRRRRR) [20,25], sHGP-9r (RGWEVLKYWWNLLQY-GG-RRRRRRRRR). The nine-arginine residues attached to the C-terminus were in D-form. I4R-9r variants were listed in Table 1. The siRNAs were obtained from Bioneer Inc (Daejeon, Korea). The sequences of siRNAs (5’-3’) are: siGAPDH (GUGGAUAUUGUUGCCAUCAdTdT), siLuciferase (GGACGAGGACGAGCACUUCUUdTdT), and control siRNA as siCONTROL (CCUACGCCACCAAUUUCGUdTdT). Fluorescein isothiocyanate (FITC) labeled siRNAs (the 5`-end of sense strand conjugated with FITC dyes) were obtained from Bioneer Inc.

### Gel retardation assay and RNase stability

Peptide-woven siRNA nanocomplexes (PwSN) were prepared by mixing siRNA in diethylpyrocarbonate (DEPC)-treated water (100 pmoles) with peptide carrier with a molar ratio of 1:5 to 1:50 (siRNA: peptide) for 30 min at room temperature. The samples were subjected on 2% agarose gels electrophoresis to examine siRNA encapsulation. To test the stability of the siRNA nanocomplex against RNase A, the nanocomplex containing 100 pmol of siRNA mixed with 20-fold molar excess of peptides, then was incubated in the presence or absence of RNase A (0.2 μg) for up to 6 h at 37°C. After incubation, the RNase A activity was inhibited by addition of 2% sodium dodecyl sulfate (SDS) and subjected to agarose gel electrophoresis [25].

### Measurement of shape, size, and surface charge

Peptide-woven siRNA nanocomplexes were prepared as described above with 1:20 molar ratio (siRNA:peptide). The hydrodynamic radii and zeta potential of nanocomplexes were determined using the zeta-potential and dynamic light scattering (DLS) instrument (ELS-Z, Otzuka Electronics, Japan). The morphology of nanocomplex was observed by transmission electron microscopy (TEM, CM30 Electron Microscope, Philips, CA). Nanoparticles deposited on the grid were negatively stained with 2 wt.% uranyl acetate solution [26].

### Cell culture and MTT assay

HeLa cells were obtained from ATCC (Manassas, VA) and cultured in Dulbecco’s modification of Eagle’s medium (DMEM) with 10% fetal bovine serum, penicillin (100 IU/ml) and streptomycin (100 (/ml) at 37°C and in the 5% CO_2_ atmosphere. Cytotoxicity of siRNA nanocomplex was assessed in HeLa cell. The cells (5×10^3^ cells/well) were seeded on 96-well plates and incubated for 24 h, after which the medium was replaced with fresh DMEM containing I4R-9r PwSN (31.25 nM to 500 nM siRNA). As control experiments, equivalent amounts of naked siRNA were added into fresh medium. After total of 48 h incubation, the cell viability was evaluated with MTT (3-[4,5-dimethylthiazol-2-yl]-2,5-diphenyl tetrazolium bromide) assay as previously described [27].

### Cell binding and uptake of siRNA nanocomplex

FITC-siRNA was mixed with 20-fold molar excess of I4R-9r tandem peptides and incubated with 1 × 10^5^ HeLa cells in suspension at 4°C for 1 hr. The cells were preincubated with 1% bovine serum albumin (BSA) at 4°C for 30 min for blocking. Cell binding and uptaking was determined using FACS Calibur flow cytometer (Becton-Dickinson, San Jose, CA). The binding specificity of I4R-9r/FITC-siRNA nanocomplexes was assessed by blocking experiments in which HeLa cells were preincubated with antibody to human IL-4R (ab50018, abcam) and IgG control antibody (ab91361, abcam) at 4°C for 1 hr. To analyze cellular uptaking of each tandem peptide, series of siRNA concentrations (31.25nM~250nM) was mixed with peptide and tested. For confocal microscopic analysis, cells were attached in eight-chamber glass slide (Lab-Tek II, Nunc, Inc., Rochester, NY), incubated with the I4R-9r/siRNA-FITC nanocomplexes for 4 h at 37°C, the nuclei were stained with DAPI, and slides were mounted and analyzed (TCS SP8, Leica, Germany) [25].

### In vitro Gene Silencing analysis

HeLa cells (1.5×10^5^) were plated 24 h prior in 35 mm dishes and allowed to reach at least 70% in confluency. Nanocomplexes were added at 200 nM siRNA/ 2uM peptide (I4R-9r/siGAPDH and I4R-9r/siControl) over the cells for 4 h at 37°C in serum-free DMEM. Cells were then washed and incubated with fresh culture medium containing 10% FBS for an additional 48 h before being lysed and examined by western blot with specific antibodies [28]. As a positive control, transfection with Lipofectamine 2000 (Invitrogen, Carlsbad, CA) was performed in accordance with the manufacturer’s instructions.

### Endosomal Escape

HeLa cells were plated 48 h prior in eight chamber slides and allowed to reach at least 70% confluence. The cells were treated with the early endosome marker (CellLight Early Endosome-RFP, Molecular Probe) for 12 h before the transfection of I4R-9r/siRNA-FITC nanocomplex [29,30]. After 4 hours after the transfection, the cells were fixed with 4% paraformaldehyde and observed under the laser scanning confocal microscope to monitor the endosomal escape of the nanocomplexes.

### In vivo gene silencing efficacy analysis

This study strictly followed the recommendations of National Institute of Health (NIH) for the Care and Use of Laboratory Animals. Animal experiments were reviewed and approved by the Committee on the Ethics of Animal Experiments of the Korea Institute of Science and Technology (KIST). All efforts were made for minimizing animal suffering. Male nude mice (BALB/c nude; body weight, 20±2 g; n = 9) were housed in a specific pathogen-free environment at 22±2°C, 55±5% relative humidity with light. Prior to the in vivo imaging, tumor bearing mice were anesthetized under inhalational anesthesia (1%, w/v, isofurane in 2 L oxygen), then D-luciferin (150mg/kg) was then injected intraperitoneally. 3 mice per group was ued to minimize unnecessary sacrifice.

To monitor the feasibility of *in vivo* gene silencing efficacy of I4R-9r(A1*)/sHGP-9r-PwSN, HT29-luc human colorectal adenocarcinoma cells (1 X 10^7^ cells) were injected subcutaneously in the left flank of mouse. After 3 weeks, mice received the I4R-9r(A1*)-PwSN (40μg of siRNA/mouse) via intra-tumoral injection every other day. Bio-luminescence signals of subcutaneous tumor were monitored using the IVIS Spectrum imaging system (Caliper Life Sciences, Hopkinton, MA) during the experiment period.

### Heparan sulfate competition assay

After preparing I4R-9r-PwSN (siRNA 1 μg), different amounts of heparan (0, 5, 10, 20 and 40 μg) were added to the solution and further incubated for 30 min. The released amount of siRNA by heparan sulfate was analyzed by 2% agarose gel electrophoresis and visualized by staining with EtBr [31].

### Surface plasmon resonance (SPR) analysis

The dynamics of PwSN formation were studied by SPR analysis (SR7500 DC, Reichert Inc, Depew, NY). 5′-Biotinylated siRNAs were diluted to 1 μM in HBS buffer (10 mM HEPES, pH 7.4, 150 mM NaCl, 3.4 mM EDTA, 0.005% Tween20), heated at 85°C for 10 min, cooled to room temperature, diluted to 20 fmol/μl in running buffer (10 mM HEPES, pH 7.4, 150 mM NaCl, 62.5 μg/ml bovine serum albumin, 125 μg/ml tRNA, 1 mM dithiothreitol, 0.05% Tween20), and injected at 10 μl/min. 50–100 response units of siRNA were coated per flow channel on streptavidin-coated sensor chip (NeutrAvidin Surface chip; Reichert Analytical Instrument, Depew, NY). 2-min injections of four different peptide concentrations (10, 5, 2.5, and 1.25 μ M) were injected over the sensor chip surface at 25°C with a flow rate of 25 μl/min. The surface was regenerated by removing bound peptides with a 180-s 2 M NaCl injection and 20-s 0.1% SDS followed by a binding buffer injection to rinse the needle. Sensorgrams were fit to kinetic titration model using Clamp program. To monitor dissociation between peptide and siRNA upon heparan sulfate competition, The 2.5 μM of peptides were injected to reach saturated binding and different concentrations of heparan sulfate were injected.

### 3D structure models of I4R-9r tandem peptides

The sequence of each I4R-9r variant was submitted to PEPFOLD server for peptide folding including PSIPRED prediction. PEP-FOLD is a *de novo* approach aimed at predicting peptide structures from amino acid sequences. The lowest energy model was visualized using PyMOL Molecular Graphic System (Version 1.2, Schrodinger, LLC) after energy minimization using Chimera (http://www.cgl.ucsf.edu/chimera) [32–35].

### Statistical analysis

Data were presented as mean ± standard deviation. Statistical significances were determined using the ANOVA analysis and Student`s *t*-test for pairwise comparison. The significance was accepted for *P* values < 0.05.

## Results

### Tumor-specific cellular uptake of I4R-9r PwSN

**Fig 1:**
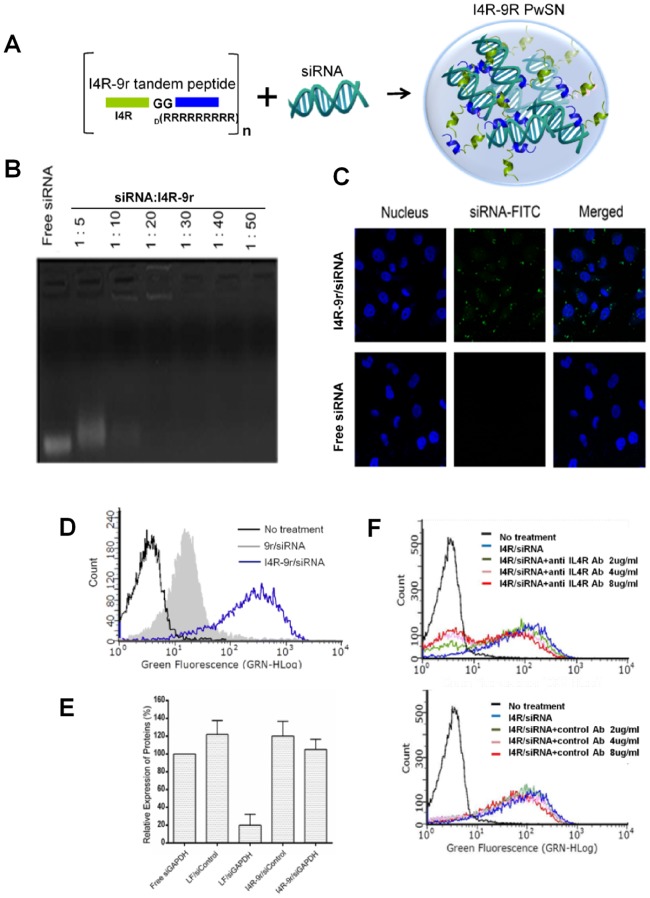
Design, biochemical characterization, and gene silencing activity of I4R-9r PwSN. (A) Schematic representation of the Peptide-Woven SiRNA Nanocomplex (PwSN), with siRNA noncovalently bound to I4R-9r tandem peptides composed of a tumor targeting peptide (I4R, green) and Cell penetrating peptide (9R, blue) separated by a 2-glycine spacer. (B) siRNA encapsulation by I4R-9r tandem peptides was monitored by gel retardation assay with molar ratios of 1:5 to 1:50 (siRNA-to-peptide). (C) Representative confocal microscopy images of HeLa cells treated with siRNA-FITC carried I4R-9r PwSN *vs*. free siRNA-FITC. (D) Representative histograms from flow cytometry for cellular uptake of I4R-9r PwSN (blue), 9r PwSN (grey), and free siRNA (black). (E) Representative histograms from flow cytometry for cellular uptake of I4R-9r PwSN (blue) vs. free siRNA (black) in the presence of indicated concentrations of anti-IL4R antibody or an IgG control. (F) HeLa cells were transfected with nanocomplexes carrying siRNA against GAPDH. The GAPDH protein expression is monitored by Western blot and presented with mean of relative immunoblot intensities. Lipofectamine was used as a positive control. The error bars represent SD from cumulative data of six independent experiments.

To develop a tumor-specific siRNA delivery system, we synthesized a tandem peptide carrier composed of an N-terminal tumor targeting I4R peptide (CRKRLDRNC) and C-terminal 9 D-arginine repeats (Fig. 1A). The I4R peptide homes to tumors through specific binding to the IL-4 receptor, which is expressed on the surface of tumor cells [20,25]. To determine the amount of peptide needed to fully encapsulate free siRNAs into nanocomplexes, we mixed siRNA with tandem peptides at increasing molar ratios (Fig. 1B). Gel retardation assays showed that, upon nanocomplex formation, free siRNA decreased and most of the siRNA was encapsulated by I4R-9r at siRNA:peptide molar ratios between 1:20 and 1:50. The biocompatibility of the siRNA nanocomplex was evaluated by testing cytotoxicity toward HeLa cells using MTT assay. No significant decrease in cellular viability was observed, indicating that I4R-9r PwSN was not toxic at the concentrations examined (Figure A in S1 File). To gain insight into the ability of I4R-9r PwSN to deliver siRNA, we assessed intracellular uptake of siRNA, the first critical barrier in siRNA delivery, using confocal microscopy and flow cytometry (Fig. 1C and D). The FITC-conjugated siRNA was encapsulated by I4R-9r tandem peptides, forming I4R-9r PwSN, and incubated with HeLa cells. Fluorescent siRNAs in the nanocomplex were observed in the cytoplasm of HeLa cells, whereas no signals of free siRNA were detected, indicating the I4R-9r tandem peptides efficiently delivered fluorescent labeled siRNA into the cytosol (Fig. 1C). A FACS analysis also showed specific cellular uptake of I4R-9r PwSN; this uptake appeared to be reinforced by specific interaction of the I4R peptides with IL-4 receptors since I4R-9r PwSN exhibited stronger binding to HeLa cells than 9r-PwSN (Fig. 1D). To determine whether the cellular uptake of I4R-9r PwSN was mediated by direct interaction with the IL4 receptor, we performed blocking experiments using a series of concentrations of an anti-IL-4R antibody (Fig. 1E). Preincubation with an anti-IL-4R antibody specifically diminished cellular uptake in a concentration-dependent manner compared to a control antibody, suggesting that, because of the presence of I4R, the tandem presentation of 9R leads to specific uptake by cells expressing IL-4 receptors.

We next investigated the *in vitro* gene-silencing activity of siRNAs delivered by I4R-9r PwSN. HeLa cells were treated with siRNA against human glyceraldehyde-3-phosphate dehydrogenase (GAPDH) bound to the I4R-9r tandem peptide or lipofectamine, and then analyzed for GAPDH knock down by Western blot. As shown in Fig. 1F, I4R-9r PwSN carrying siGAPDH induced a slight, but statistically insignificant, decrease in GAPDH protein expression in HeLa cells, whereas siGAPDH delivered by lipofectamine dramatically decreased GAPDH expression. The insignificant silencing effect of I4R-9r PwSN, despite its specific and effective cellular targeting, prompted us to develop strategies for overcoming barriers after entry of the nanocomplex into cells.

### sHGP-9r tandem peptide for endosomal escape

**Fig 2:**
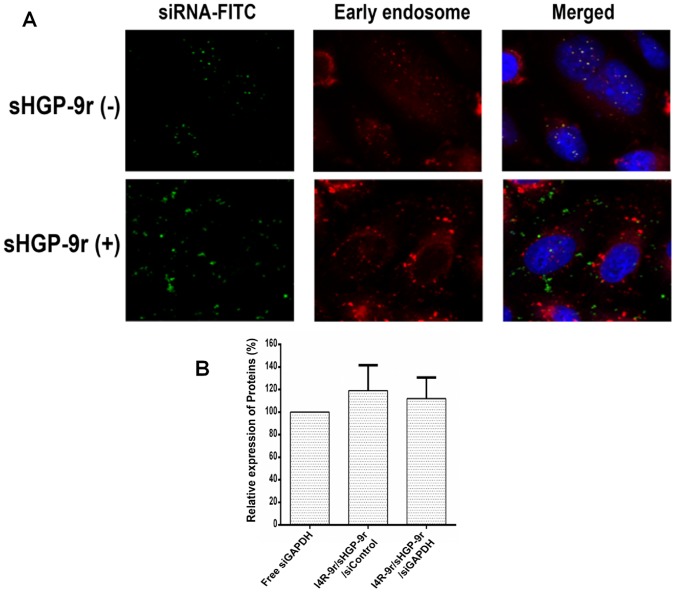
Addition of sHGP-9r to facilitate endosome escape of nanocomplexes. (A, Top) Confocal microscopy images of HeLa cells pretreated with the early endosome marker-RFP, and subsequently incubated with I4R-9r PwSN carrying FITC labeled siRNA. Images were pseudocolored for visualization: blue = DAPI; red = early endosome marker-RFP; green = FITC-siRNA. (A, Bottom) Confocal microscopy images of HeLa cells after treatment with FITC-siRNA encapsulated in I4R-9r PwSNs, which compose of sHGP-9r at molar ratio of 1:19 (sHGP-9r: I4R-9r). (B) The gene silencing efficacies against GAPDH protein with or without the sHGP-9r peptides was assessed by Western blot. Results are presented as mean of relative immunoblot intensities ± standard deviation (n = 6).

After cellular internalization of the nanocomplex via receptor-mediated endocytosis, the next critical step in the delivery process is escape from the early endosome [11,36–38]. Fluorescent siRNAs delivered by I4R-9r PwSN were present in punctuate vesicular structures, consistent with sequestration in endosomes (Fig. 1C). To directly confirm the intracellular localization of siRNAs, we applied nanocomplexes to HeLa cells pretreated with the early endosome marker-RFP and monitored FITC-conjugated siRNA by confocal microscopy (Fig. 2A). Fluorescently labeled siRNA colocalized with the early endosome marker, suggesting that I4R-9r PwSN carried siRNA payloads into the cells via endocytosis and was initially sequestered in endosomes.

To improve the efficiency of endosomal escape, we synthesized the endosomolytic tandem peptide, sHGP, linked it to 9r (sHGP-9r), and blended it with I4R-9r PwSN at a 1:19 molar ratio (sHGP-9r- to- I4R-9r). When the multicomponent PwSN (I4R-9r/sHGP-9r) was applied to HeLa cells, the fluorescence of FITC-conjugated siRNA no longer overlapped with the early endosome marker, indicating that this multicomponent PwSN was able to escape from the early endosomes (Fig. 2A, lower panel). However, although introduction of sHGP-9r facilitated endosomal escape, it did not fully overcome the limited *in vitro* gene-silencing efficacy of I4R-9r/sHGP-9r PwSN (Fig. 2B).

### Design and characterization of I4R-9r variant PwSNs

**Fig 3:**
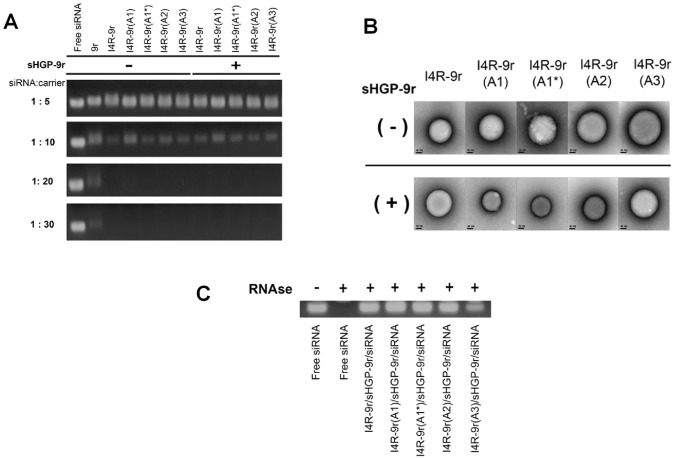
Physicochemical characterization of I4R-9r variants PwSNs. (A) siRNA encapsulations by I4R-9r variants were monitored by gel retardation assay with molar ratios of 1:5 to 1:30 (siRNA: carrier) in the presence and absence of sHGP-9r. (B) Representative TEM of an I4R-9r tandem peptide variants/siRNA nanocomplex formed in water in the presence and absence of sHGP-9r; scale bar = 50 nm. (C) Stability of I4R-9r variant/sHGP-9r/siRNA nanocomplex was examined in the presence of RNase A. Undegraded siRNA of the nanocomplex was visualized on 2% agarose gel containing EtBr. The stability of free siRNA was measured as control.

To achieve gene knockdown, the siRNA cargo must ultimately dissociate from the carrier after escaping from the endosome to allow the incorporation of siRNA into the RISC machinery. Thus, we focused on siRNA release from the nanocomplex in the cytoplasm. It has been reported that CPPs with long poly-arginine peptides (12R-15R) do not readily release siRNA, likely owing to the large number of positively charges, which retard the unpacking of siRNA [13]. In our case, the siRNA complex was formed mostly by electrostatic interactions between the positively charged 9r and negatively charged siRNA. We thus modified 9r by replacing selected arginines with alanines, which would be expected to alter electrostatic interactions. We designed and created four tandem peptide candidates—I4R-9r(A1), I4R-9r(A1*), I4R-9r(A2) and I4R-9r(A3) (Table 1)—that differed with respect to the number and position of alanine substitutions. We then formed I4R-9r-variant PwSNs and validated their biochemical characteristics as well as *in vitro* gene-silencing efficacy compared with that of I4R-9r PwSN. First, we tested the encapsulation efficiency of the I4R-9r tandem peptide variants using gel-retardation assays. As was the case with the I4R-9r peptide, all variants were able to encapsulate siRNA at molar ratios of 1:20 and 1:30. Complex formation was not affected by the presence or absence of the sHGP-9r peptide (Fig. 3A). The physicochemical characteristics of I4R-9r-variant PwSNs (1:20 siRNA-to-peptide molar ratios) were not significantly changed by the variation of 9r. Using TEM and dynamic light scattering (DLS), we observed that all I4R-9r variants assembled with siRNA to form PwSNs with a round structure around 200 nm in diameter, which is the typical size of a CPP-based siRNA nanocomplex (Fig. 3B and Table 1). Zeta potentials ranged from approximately +15 to +20 mV. The presence of sHGP-9r in the PwSN slightly reduced size and surface charge in comparison with those of sHGP-9r-free PwSNs (I4R-9r). Moreover, siRNAs carried by I4R-9r-variant PwSNs were protected from RNases A for at least 6 h at 37°C, whereas free siRNA was completely degraded (Fig. 3C). One of the variant PwSNs, I4R-9r (A3), in which three arginines were substituted with alanines, showed 50% protection of its siRNA payload. FACS analysis of the cellular uptake of I4R-9r-variant PwSNs containing different concentrations of siRNA (31.25 to 250 nM) showed that, with the exception for I4R-9r(A3), more than 80% of siRNA was transferred into cells by nanocomplexes at siRNA concentrations greater than 100 nM (Figure B in S1 File).

**Table 1:** Structural Properties of I4R-9r/sHGP-9r PwSN variants.

*PwSN name*	*sequence* ^*a*^	*diameter (nm)* ^*b*^	*ζ-potential(mV)* ^*c*^
I4R-9r	CRKRLDRNCggRRRRRRRRR	197.7±38.8	27.35±5.03
I4R-9r/sHGP-9r	CRKRLDRNCggRRRRRRRRR/sHGP-9r	161.0±6.36	18.22±2.06
I4R-9r(**A1**)/sHGP-9r	CRKRLDRNCggRRRR**A**RRRR/sHGP-9r	163.8±1.03	17.83±5.96
I4R-9r(A1*)/sHGP-9r	CRKRLDRNCggRR**A**RRRRRR/sHGP-9r	149.7±2.26	15.50±3.38
I4R-9r(**A2**)/sHGP-9r	CRKRLDRNCggRR**A**RRR**A**RR/sHGP-9r	174.1±1.06	19.60±0.16
I4R-9r(**A3**)/sHGP-9r	CRKRLDRNCggR**A**RR**A**RR**A**R/sHGP-9r	177.1±2.09	14.33±6.72

^*a*^ sHGP-9r (RGWEVLKYWWNLLQYggRRRRRRRRR) is blended with I4R-9r/sHGP-9r PwSN with 1:19 molar ratios (sHGP-9r: I4R-9r). gg; Gly-Gly linker. Substituted alanine was highlighted by red.

^*b*^ Mean hydrodynamic size based on dynamic light scattering measurements. Errors indicate SD from at least three separate measurements.

^*c*^ Zeta-potential of nanocomplexes. Errors indicate SD from at least three independent measurements.

### Gene-silencing efficacy of I4R-9r(A1*)/sHGP-9r PwSN

**Fig 4:**
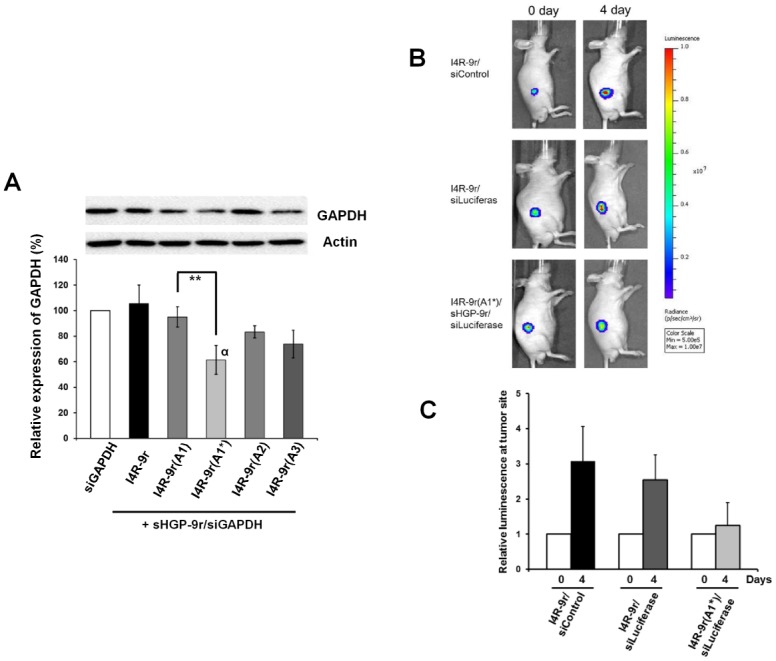
I4R-9r variant PwSNs mediated gene silencing *in vitro* and *in vivo*. (A) The relative amount of GAPDH knockdown was determined by Western blot. The intensities of immunoblot was normalized with those of beta actin control and results are presented as mean ± standard deviation (n = 5, (α) ANOVA test, P<0.05: (_**_) t-test, P<0.01). (B) To evaluate *in vivo* gene silencing efficacy, The HT29-luc bearing mice were treated with I4R-9r/siControl, I4R-9r/siLuciferase and I4R-9r(A1*)/sHGP-9r/siLuciferase PwSNs via intra-tumoral injection. Bio-luminescence signals of subcutaneous tumor were monitored using the IVIS Spectrum imaging system in day 0 and 4. (C) The bioluminescence intensities at tumor site were obtained and the relative intensities were calculated based on the bioluminescence intensities of each group in day 0 as 1. Error bar represents SD from three independent results.

We next investigated the *in vitro* gene-silencing activity of siRNAs delivered by I4–9r-variant PwSNs against the target GAPDH (Fig. 4A). Most of I4R-9r-variant PwSNs knocked down GAPDH expression with varying and insignificant degrees of efficacy compared with I4R-9r PwSN. However, I4R-9r(A1*)/sHGP-9r PwSN induced a statistically significant, 42% suppression of GAPDH expression. Thus, I4R-9r(A1*)/sHGP-9r PwSN was selected as an optimized carrier candidate and tested for effective gene-silencing in an animal model system.

Prior to animal experiments, the gene-silencing activity of I4R-9r (A1*) PwSN against luciferase was tested in HT29-luc cells, which stably express luciferase (Figures C and D in S1 File). I4R-9r (A1*) PwSN was efficiently taken up by HT29-luc cells and exhibited gene-silencing activity by reducing luciferase proteins by ~52%. We next developed a mouse xenograft model using these HT29-luc cells. The bioluminescence signals of the tumor were compared before and after intra-tumoral injection of the nanocomplex. As shown Fig. 4B and C, luminescent signals in tumors of control mice increased approximately 3-fold after 4 d, whereas those of the I4R-9r(A1*) PwSN-treated mice increased by only 1.25-fold, suggesting that the siRNA carried by I4R-9r(A1*) PwSN successfully silenced its specific target *in vivo*. The siRNA carried by the unmodified I4R-9r PwSN exhibited marginal activity, consistent with its insignificant effect *in vitro*.

### Dynamic interactions and structures of I4R-9r-variant/siRNA nanocomplexes

**Fig 5:**
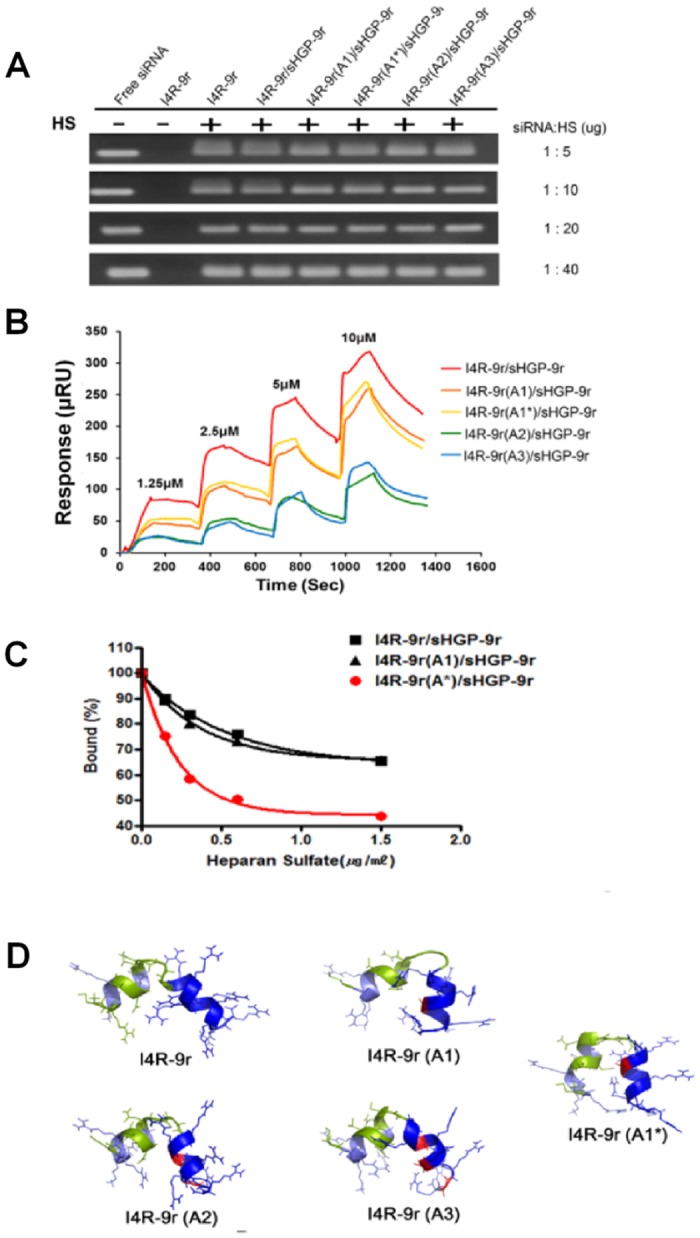
Dynamic interactions and structures of I4R-9r variant/siRNA nanocomplexes. (A) The released siRNA from nanocomplexes were examined upon addition of excess amount of Heparan sulfate (w:w of siRNA to Heparin sulfate as 1:5–1:40)using gel retardation assay. (B) The kinetics of association of siRNA with I4R-9r variant/sHGP-9r using SPR analysis. Increasing concentrations of I4R-9r variant/sHGP-9r peptides were injected to associate with 5`-biotinylated siRNA on the streptavidin chip. (C) The kinetics of disassociation of siRNA with I4R-9r variant/sHGP-9r using SPR analysis. After association of 5`-biotinylated siRNA with I4R-9r variant/sHGP-9r (2.5 μM) up to saturation different concentrations of heparan sulfate were injected to quantitate siRNA dissociation. The dissociation kinetics was analyzed using the Graph Prism 5.0. (D) The structures of I4R-9r variant were analyzed and compared by 3D modeling method (PEPFOLD); I4R peptide (green), Arginine of 9r (blue), Alanine (red).

One possible explanation for the improved efficiency of I4R-9r(A1*) PwSN despite the shared biochemical characteristics of I4R-9r variant PwSNs described above involves the rate of nanocomplex dissociation. To test this, we assessed the dissociation of siRNA from nanocomplexes by performing heparan sulfate competition assays using gel-retardation assays. The encapsulated siRNAs began to dissociate from their corresponding nanocomplex upon addition of more than a 5-fold excess of heparan sulfate; siRNA release was similar for all variants except I4R-9r(A3) PwSN, which showed increased release of siRNA (Fig. 5A). To further explore the dynamic interactions of each carrier with siRNA, we examined the kinetics of I4R-9r tandem peptide variant binding with siRNA using SPR analysis. In these experiments, I4R-9r peptide variants with sHGP-9r (19:1 molar ratios) were injected at increasing concentrations onto immobilized siRNA, and association and dissociation were measured (Fig. 5B). The I4R-9r tandem peptide showed the strongest binding affinity for siRNA, presumably because of its higher number of positive charges. The single alanine-substituted variants, I4R-9r(A1) and I4R-9r(A1*), showed moderate affinity, and the double and triple alanine-substituted variants, I4R-9r(A2) and I4R-9r(A3), respectively, showed the lowest affinity, as expected by their diminished overall positive charge. In the end, we selected the most efficient binding candidates, I4R-9r, I4R-9r(A1) and I4R-9r(A1*), and tested their dissociation from siRNA in the presence of excess heparan sulfate (Fig. 5C). Heparan sulfate induced a concentration-dependent dissociation of siRNA from I4R-9r-variant PwSNs, providing a possible indication of complex disassembly in the presence of anionic components in the cytosol. Interestingly, siRNA dissociated more rapidly from I4R-9r(A1*) PwSN than from the other variants, suggesting that the improved gene-silencing activity of I4R(A1*) PwSN is attributable to rapid dissociation of siRNA in the cytosol. Similar results were observed for nanocomplexes lacking sHGP-9r (Figures E and F in S1 File). To understand why I4R-9r(A1*) PwSN was more efficient in unpacking siRNA than I4R-9r(A1) and other variants, we investigated the molecular structures of I4R-9r-variant tandem peptides using 3D modeling (Fig. 5D). The 3D structural models showed that the I4R-9r(A1*) tandem peptide adopted a “U”-shaped structure, whereas other tandem peptides adopted an “L”-shaped structure. Thus the structural changes induced by the position of alanines could affect the efficiency of nanocomplex unpacking, which may in turn affect the availability of siRNA within the cytosol and contribute to RNAi-mediated gene silencing.

## Discussion

**Fig 6:**
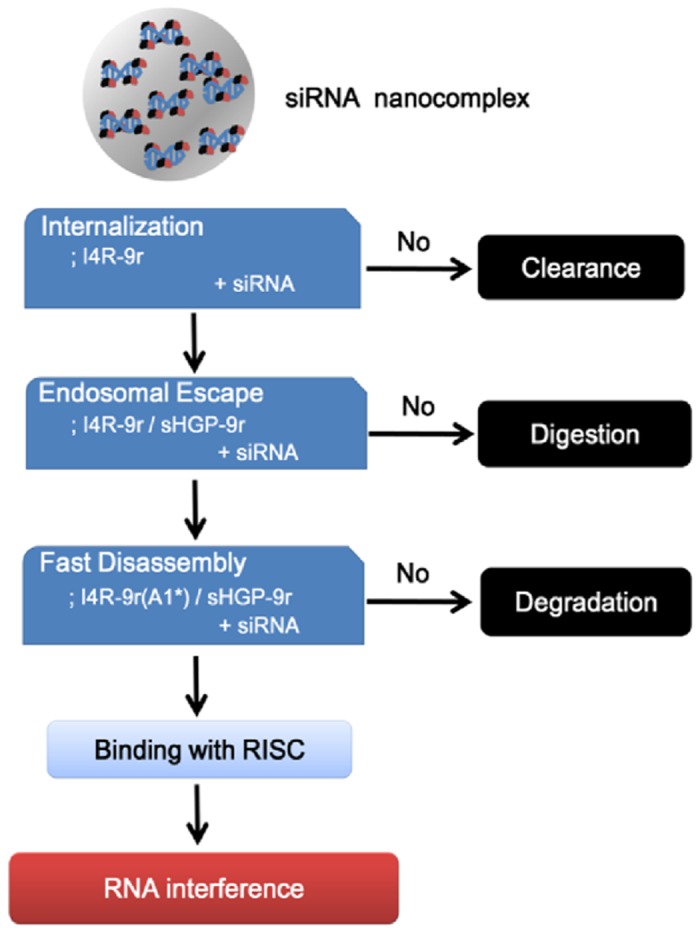
Flowchart of developing multicomponent PwSN by stepwise approach to overcome biological barriers of siRNA delivery.

To trigger RNAi activity, siRNA molecules must be delivered to the interior of specific cells and become loaded into RISCs in the cytosol. Although the mechanistic understanding of the delivery process is limited, there are general guidelines for optimal delivery systems. An optimized siRNA carrier is one that is able to assist specific uptake by target cells and overcome intracellular barriers posed by escape from endosomes or lysosomes and liberation of siRNA from nanocomplexes in the cytoplasm [10–12]. In this study, we started with a simple 9r-CPP to encapsulate siRNA, and then implemented a sequential trouble-shooting strategy to resolve problems encountered in the siRNA-delivery process, ultimately developing an optimized multicomponent PwSN with specific and effective gene-silencing ability (Fig. 6).

Arginine- or lysine-rich CPPs efficiently encapsulate siRNA through electrostatic interactions and form complexes of approximately 200 nm in diameter, which is an ideal nanoparticle size: large enough to avoid renal filtration, but small enough to evade phagocytic clearance [39]. The main drawback of CPP-based siRNA carriers is their lack of target specificity. To enhance the specificity of CPPs, we fused the tumor-homing peptide, I4R, to 9r-CPP [20]. Results obtained in other delivery systems have shown that endogenous targeting ligands are often beneficial for improving uptake by specific cells [25]. Efficient targeting may also diminish the chance of nonspecific interaction with serum proteins, including nucleases, as well as non-targeted cells.

After specific targeting and subsequent receptor-mediated endocytosis, I4R-9r PwSN was sequestered within endosomes. Generally, siRNA complexes in early endosomes are transferred to the late endosome and then to the lysosome, where various nucleases ultimately degrade the siRNA. Technologies for promoting endosomal release have recently been developed, including the use of fusogenic lipids, polymers with high buffering capacity, and membrane-interacting peptides [19,37,38,40]. To resolve the endosome entrapping problem, we blended a small amount of an endosomolytic tandem peptide (sHGP-9r) with I4R-9r. Addition of the sHGP-9r peptide facilitated endosomal escape of nanocomplexes in the cytosol, likely by imparting of the lipophilicity of the nanoparticle. The slight reduction in the size and charge of PwSN containing an HGP-9r tandem peptide can be explained by improved lipophilicity.

Achieving gene-silencing activity ultimately requires that the siRNA cargo be liberated from the delivery complex after escaping from the endosome to allow the incorporation of siRNA into the RISC machinery. Since the positively charged, arginine-rich CPP binds the negatively charged backbone of the siRNA, the efficacy of siRNA delivery can be enhanced and sustained by changing the number of arginines in the CPP [38–40]. In an effort to improve siRNA unpacking, we modified the number and positions of the positive charges in the 9r domain in the tandem and analyzed complex stability in the presence of heparan sulfate using SPR spectroscopy. Addition of heparan sulfate mimics encounters with polyanionic molecules in the cytosol. Among I4R-9r-variant PwSNs, I4R-9r (A1*) PwSN, which most efficiently liberated siRNA, exerted a significant silencing effect both *in vitro* and *in vivo*. Our results indicate that the peptide charges affect the efficiency of nanocomplex unpacking and in turn contribute to RNAi-mediated gene silencing. The less positively charged 9r variants, such as A2 and A3, exhibited poor binding and encapsulating capability compared with more highly charged 9r variants, likely because the fewer cationic charges available for binding the negatively charged siRNA reduces the strength of the interaction with siRNA and provides insufficient charge shielding for membrane translocation. However, strong binding affinity between the siRNA and its carrier is a double-edged sword: it is advantageous in that it confers a high encapsulating capacity and shields siRNA during transport to target cells, but it may also prevent the release of siRNA in the cytosol [1,11]. Accordingly, in designing the carrier, we must consider not only complex assembly in the extracellular space but also complex disassembly in the intracellular space [11]. The degree of complexation between siRNA and carrier is determined by multiple factors, including electrostatic interactions, van der Waals forces, and nonpolar salvation [11,41,42]. Our data suggest that SPR analyses can be used to reveal the dynamics of siRNA complexation with a resolution not possible using gel-retardation assays.

The optimized multicomponent nanocomplex, I4R-9r(A1*)/sHGP-9r PwSN, had a diameter of ~150 nm, was stable against RNase A treatment, was specifically internalized into target cells, escaped from the endosome, and efficiently liberated RNA to initiate the silencing pathway. An interesting feature of this complex is the unique tandem peptide structure of I4R-9r (A1*) revealed by a computer structural model. The “U”-shape of the I4R-9r(A1*) peptide could affect dynamic interactions with siRNA, suggesting that differences in the 3D structure of this peptide carrier could be an important contributor to its rapid dissociation and thus enhanced biological activity. The conclusion is consistent with recent reports indicating that carrier structure is a critical parameter in determining nanocomplex formation, although the delivery materials were different in these previous studies [41,43,44].

A comprehensive understanding of the molecular properties of the siRNA nanocomplex is important in designing an effective siRNA delivery system. As our understanding of the molecular properties of nanocomplexes grows, new criteria for efficient delivery systems will likely emerge. Recent studies of CPP-siRNA complexes also indicate that a comprehensive understanding of molecular properties is essential for more accurately predicting the gene-silencing effects [45]. Our sequential trouble-shooting strategy and observations of the dynamic characteristics of complexes based on binding affinity and molecular structure could provide useful guidelines for designing future multicomponent peptide-delivery vehicles. Additional studies will be required to overcome remaining challenges, such pharmacokinetics and evasion of the immune system, to enable clinical applications of this multicomponent PwSN.

## Supporting Information

S1 File Figure A. **The Cytotoxicity of I4R-9r PwSN**. The concentrations of siRNA ranged from 31.25 nM to 500 nM were treated onto the HeLa cells. After 48 hrs, the cell viability was evaluated with MTT assay. **Figure B. Cellular uptake of I4R-9r variant PwSNs**. Uptake by HeLa cells with different concentrations of siRNA (31.25 to 250 nM) was assessed by flow cytometry for I4R-9r variant PwSNs. **Figure C. The specific cellular uptake of I4R-9r.siRNA nanocomplex in HT 29-luc cells**. HT 29-luc cells were incubated with nanocomplexes carrying siRNA labeled with FITC. Uptake by HT 29-luc cells was measured by FACS for free siRNA (black line), 9r/siRNA (blue line) and I4R-9r/siRNA nanocomplex (red line). **Figure D. The *in vitro* gene silencing efficiency of I4R-9r.siRNA nanocomplex in HT 29-luc cells**. In vitro gene silencing efficacy was quantified by the Luciferase assay. Error bars represent SD from cumulative data of three independent experiments. **Figure E. SPR analysis of I4R-9r variant and siRNA in the absence of sHGP-9r**. The kinetics of association of siRNA with I4R-9r variant using SPR analysis. Increasing concentrations of I4R-9r variants were injected to associate with 5`-biotinylated siRNA on the streptavidin chip. **Figure F. Dissociation of I4R-9r variant and siRNA in the absence of sHGP-9r**. The kinetics of disassociation of siRNA with I4R-9r variant using SPR analysis. After association of 5`-biotinylated siRNA with I4R-9r variants (2.5 μM) different concentrations of heparan sulfate were injected to analysis the dissociation kinetics.
(DOCX)
