# Supplementary figures and images for: Design of a Multicomponent Peptide-Woven Nanocomplex for Delivery of siRNA
Source: PLoS One. 2015 Feb 23;10(2):e0118310. doi: 10.1371/journal.pone.0118310 (PMC4338040; doi:10.1371/journal.pone.0118310)

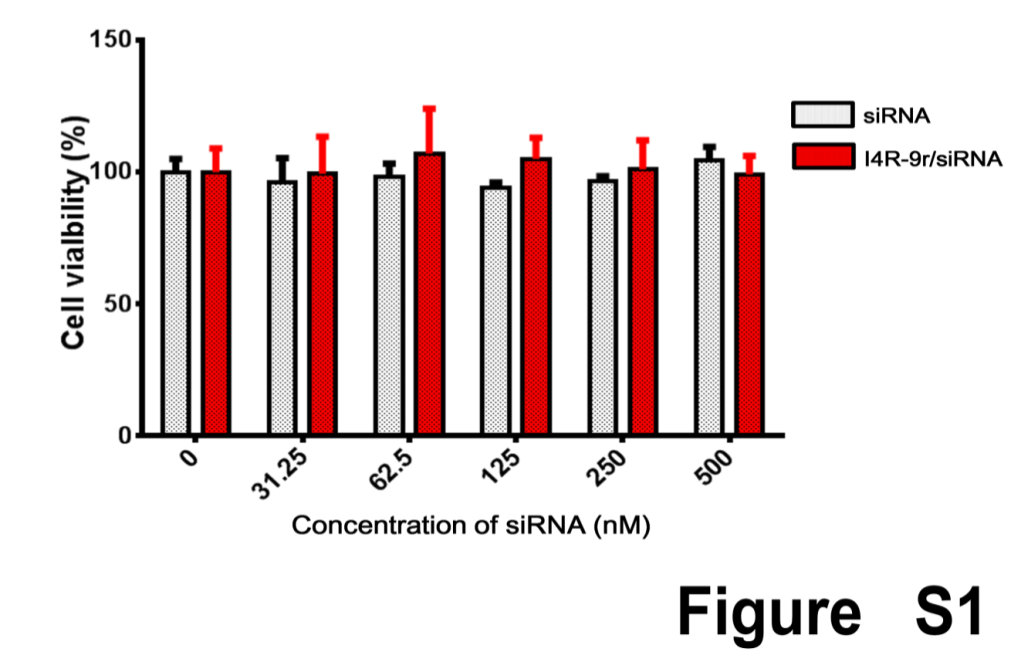


**Figure A.**


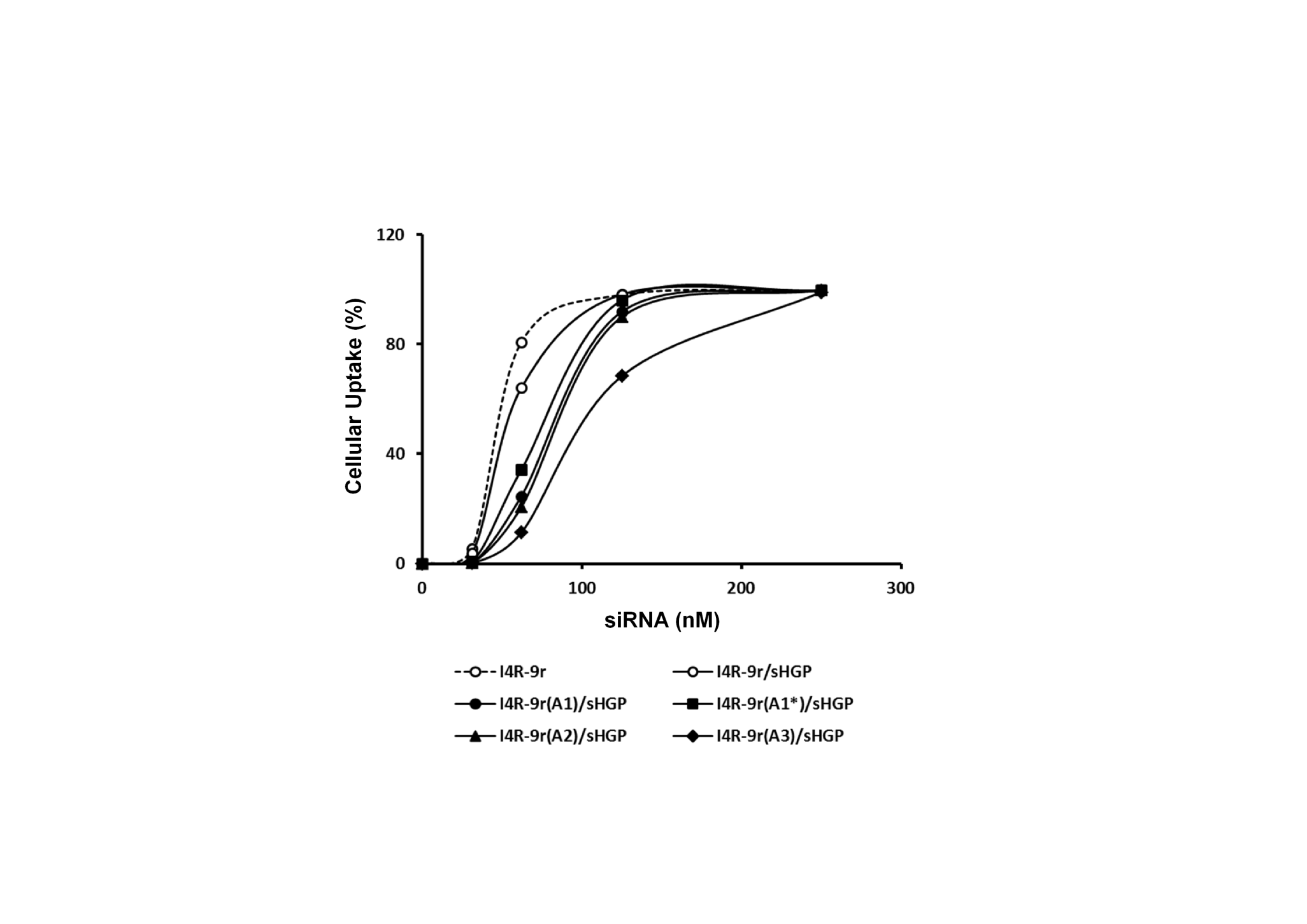


**Figure B.**


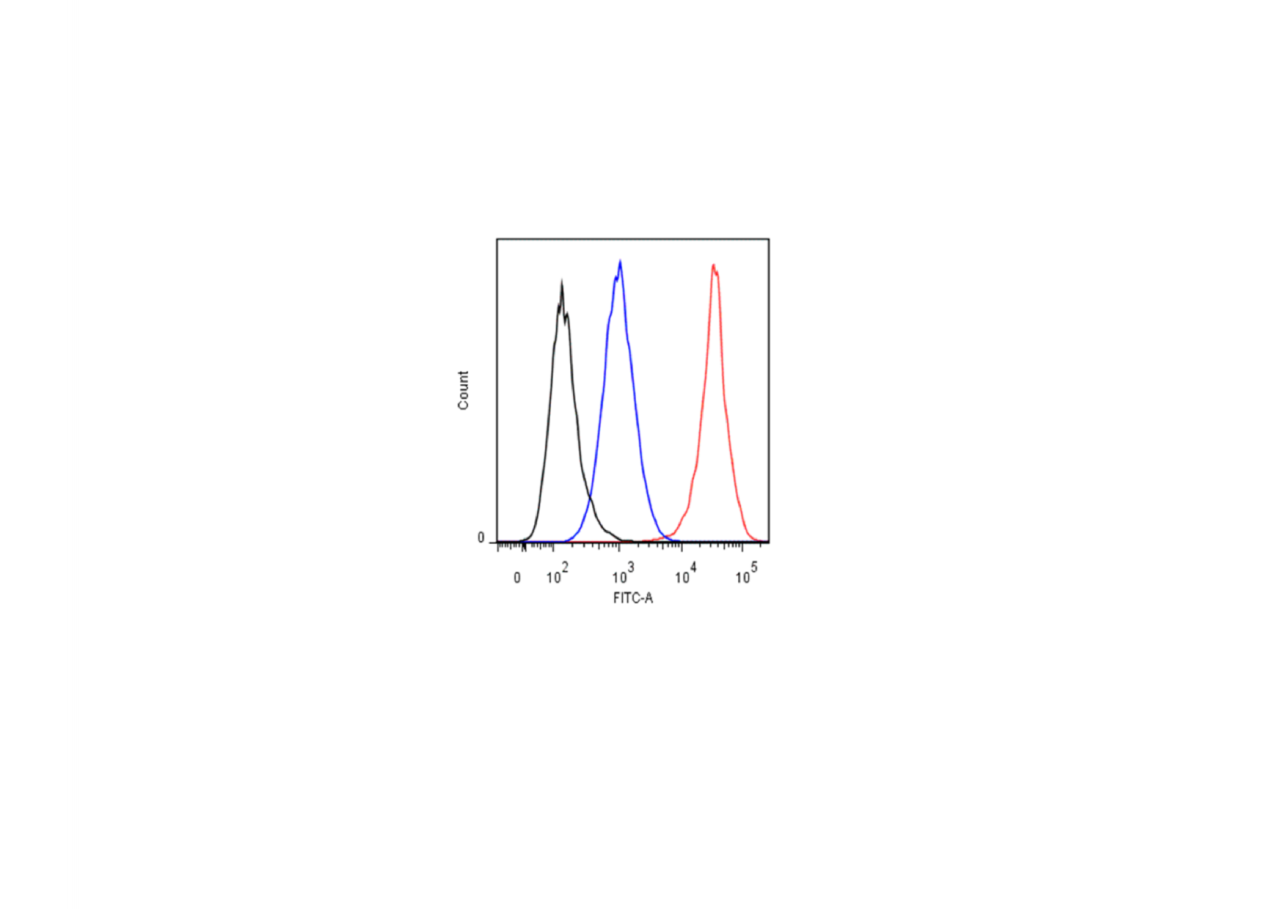


**Figure C.**


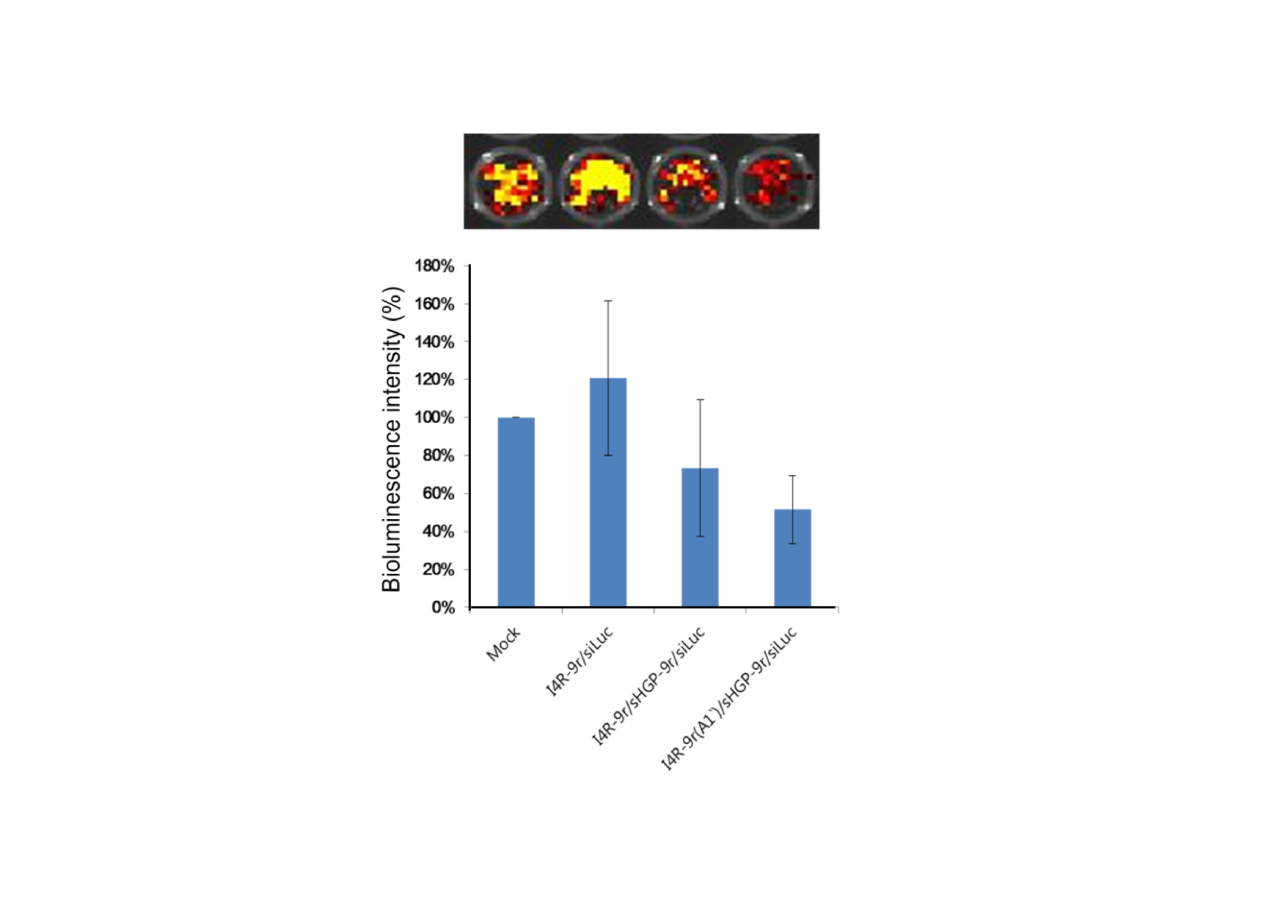


**Figure D**


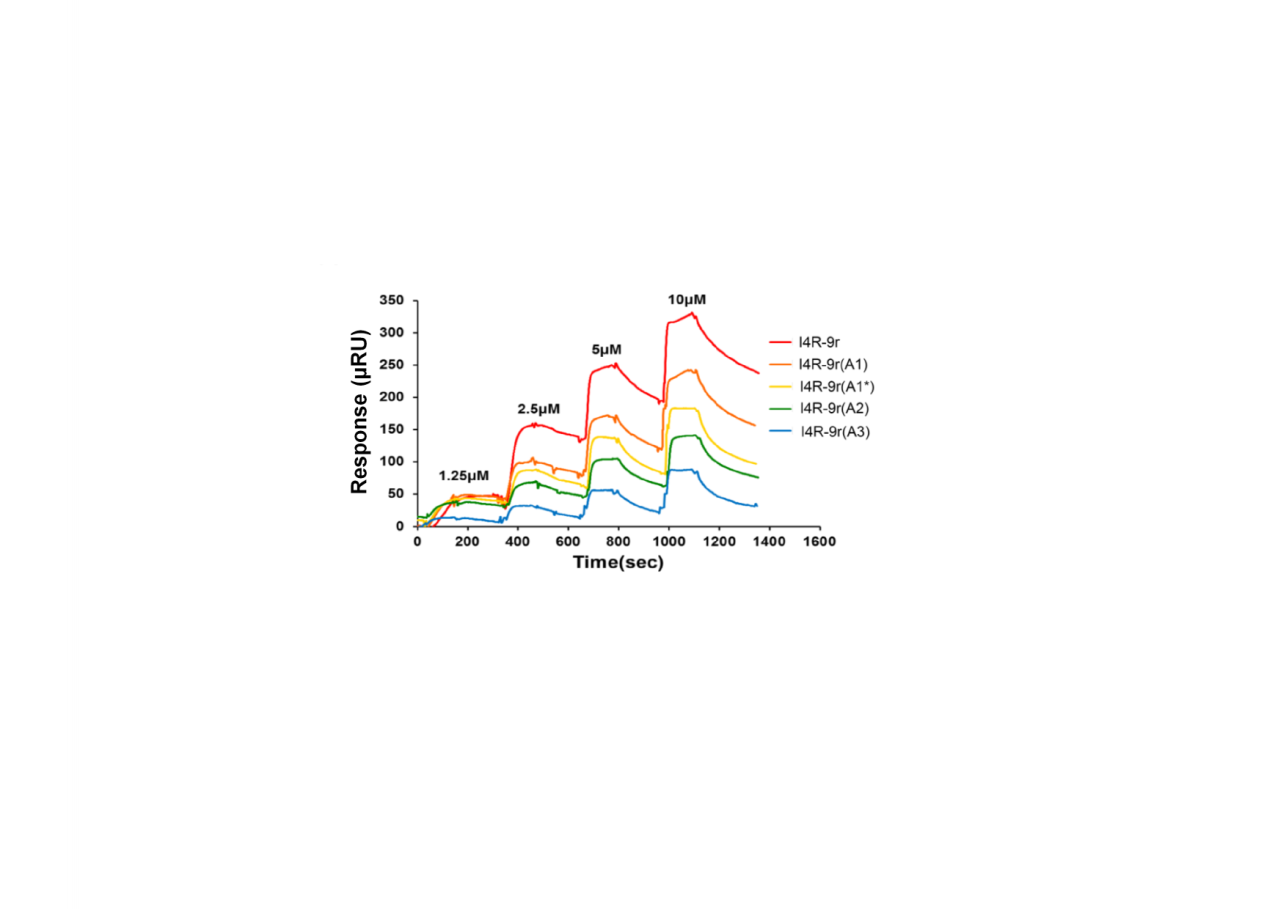


**Figure E**


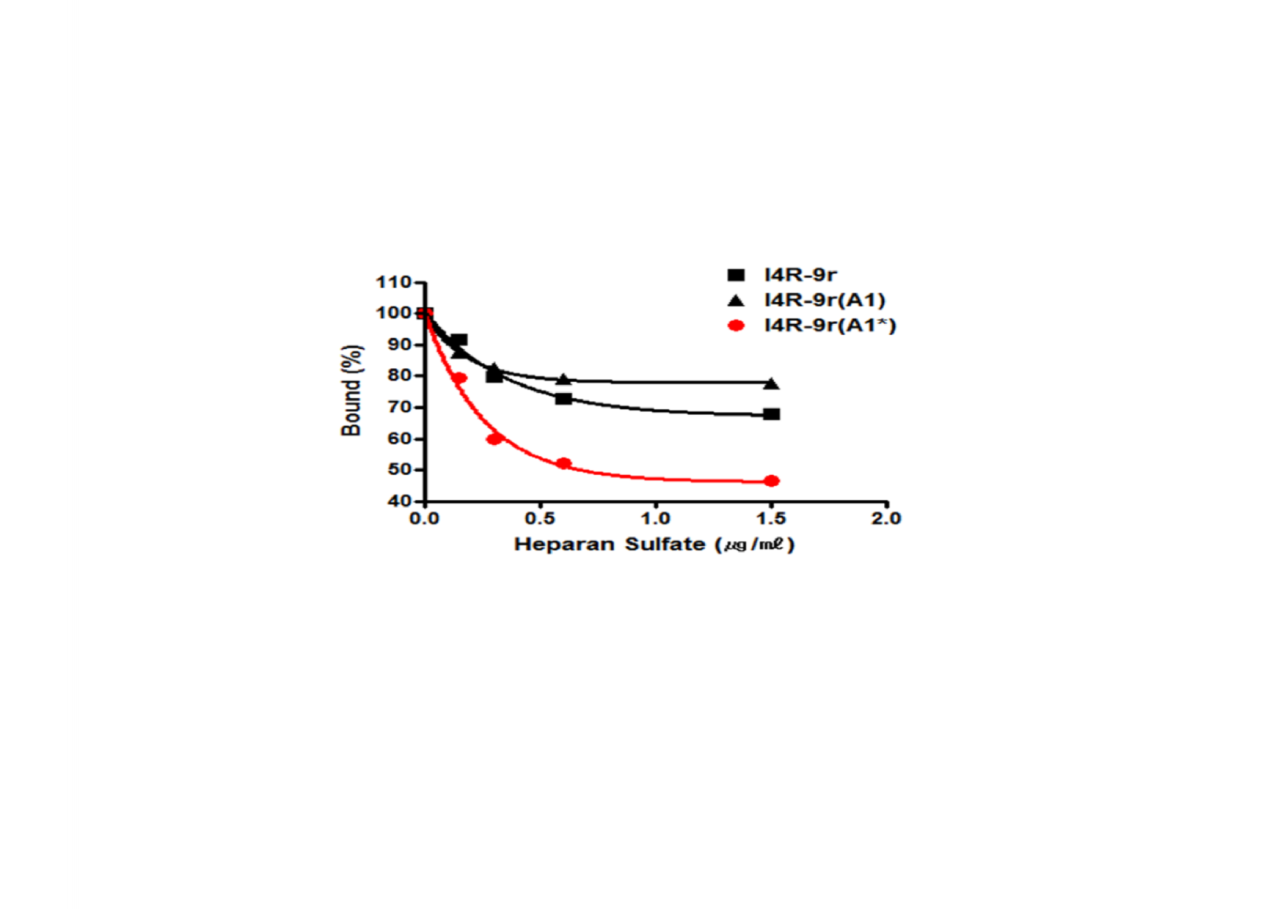


**Figure F**

Supplement: S1 File — The Cytotoxicity of I4R-9r PwSN. The concentrations of siRNA ranged from 31.25 nM to 500 nM were treated onto the HeLa cells. After 48 hrs, the cell viability was evaluated with MTT assay. Figure B. Cellular uptake of I4R-9r variant PwSNs. Uptake by HeLa cells with different concentrations of siRNA (31.25 to 250 nM) was assessed by flow cytometry for I4R-9r variant PwSNs. Figure C. The specific cellular uptake of I4R-9r.siRNA nanocomplex in HT 29-luc cells. HT 29-luc cells were incubated with nanocomplexes carrying siRNA labeled with FITC. Uptake by HT 29-luc cells was measured by FACS for free siRNA (black line), 9r/siRNA (blue line) and I4R-9r/siRNA nanocomplex (red line). Figure D. The in vitro gene silencing efficiency of I4R-9r.siRNA nanocomplex in HT 29-luc cells. In vitro gene silencing efficacy was quantified by the Luciferase assay. Error bars represent SD from cumulative data of three independent experiments. Figure E. SPR analysis of I4R-9r variant and siRNA in the absence of sHGP-9r. The kinetics of association of siRNA with I4R-9r variant using SPR analysis. Increasing concentrations of I4R-9r variants were injected to associate with 5`-biotinylated siRNA on the streptavidin chip. Figure F. Dissociation of I4R-9r variant and siRNA in the absence of sHGP-9r. The kinetics of disassociation of siRNA with I4R-9r variant using SPR analysis. After association of 5`-biotinylated siRNA with I4R-9r variants (2.5 μM) different concentrations of heparan sulfate were injected to analysis the dissociation kinetics. (DOCX) [file pone.0118310.s001.docx]
